# Post-intensive care syndrome screening: a French multicentre survey

**DOI:** 10.1186/s13613-024-01341-y

**Published:** 2024-07-09

**Authors:** Maïté Agbakou, Margot Combet, Maëlle Martin, Gauthier Blonz, Luc Desmedt, Amélie Seguin, Jérémie Lemarié, Olivier Zambon, Jean Reignier, Jean-Baptiste Lascarrou, Stephan Ehrmann, Emmanuel Canet

**Affiliations:** 1 Intensive Care Unit, Nantes University Hospital, Nantes University, 30 Bd. Jean Monnet, Nantes, Cedex 1 44093 France; 2 Intensive Care Unit, Kremlin-Bicêtre University Hospital, Assistance Publique- Hôpitaux de Paris, Le Kremlin-Bicêtre, France; 3 Intensive Care Unit, Nantes University Hospital, Movement - Interactions – Performance Research Unit (MIP, (MIP, UR 4334), Nantes, France; 4 Intensive Care Unit, Tours University Hospital, Tours, France; 5 INSERM CIC 1415, Tours University, Tours University Hospital, Tours, France; 6 Research Center for Respiratory Diseases, INSERM U110, Tours University, Tours, France; 7 Clinical Research in Intensive Care and Sepsis–Trial Group for Global Evaluation and Research in Sepsis (CRICS_TRIGGERSep), Tours, France

**Keywords:** Intensive care unit, Survey, France, Post-intensive care syndrome, Disability, Screening

## Abstract

**Background:**

Post-intensive care syndrome (PICS), defined as physical, cognitive, and mental-health symptoms persisting long after intensive-care-unit (ICU) discharge, is increasingly recognised as a healthcare priority. Data on screening for PICS are sparse. Our objective here was to describe post-ICU screening in France, with special attention to visit availability and evaluations done during visits.

**Methods:**

We conducted an online multicentre survey by emailing an anonymous 43-item questionnaire to French ICUs. For each ICU, a single survey was sent to either the head or the intensivist in charge of follow-up visits.

**Results:**

Of 252 ICUs invited to participate, 161 (63.9%) returned the completed survey. Among them, 46 (28.6%) offered follow-up visits. Usually, a single visit led by an intensivist was scheduled 3 to 6 months after ICU discharge. Approximately 50 patients/year/ICU, that is, about 5% of admitted patients, attended post-ICU visits. The main criteria used to select patients for follow-up were ICU stay and/or invasive mechanical ventilation duration longer than 48 h, cardiac arrest, septic shock, and acute respiratory distress syndrome. Among ICUs offering visits, 80% used validated instruments to screen for PICS. Of the 115 ICUs not offering follow-up, 50 (43.5%) indicated an intention to start follow-up within the next year. The main barriers to offering follow-up were lack of available staff and equipment or not viewing PICS screening as a priority. Half the ICUs offering visits worked with an established network of post-ICU care professionals, and another 17% were setting up such a network. Obstacles to network creation were lack of interest among healthcare professionals and lack of specific training in PICS.

**Conclusion:**

Only a small minority of ICU survivors received follow-up designed to detect PICS. Less than a third of ICUs offered follow-up visits but nearly another third planned to set up such visits within the next year. Recommendations issued by French health authorities in 2023 can be expected to improve the availability and standardisation of post-ICU follow-up.

**Supplementary Information:**

The online version contains supplementary material available at 10.1186/s13613-024-01341-y.

## Background

Major survival gains have been achieved by intensive care units (ICUs) in recent decades [1–3]. However, in the growing population of survivors, a syndrome of persistent ill-health is common. This post-ICU syndrome (PICS) variably combines impairments in the physical (ICU-acquired weakness, chronic organ failures, dysphagia, oral injuries, pressure injuries, malnutrition, pain, fatigue…), psychological (anxiety, depression, and post-traumatic stress disorder [PTSD]), and cognitive spheres [4–17]. Patients with PICS experience impairments in quality of life, self-sufficiency, and work ability, as well as an increased risk of death [7, 18–21]. PICS is a recently described entity whose frequency remains unclear, notably given the usual absence of pre-ICU data and variability in screening practices [22]. In a prospective cohort, 64% and 56% of the 406 patients had at least one PICS symptom 3 and 12 months after discharge, respectively, and the corresponding proportions for symptoms in at least two of the three spheres were 25% and 21%, respectively [13]. Many other studies have found high prevalence of PICS symptoms [12, 23–25]. Reported risk factors include pre-existing features (older age, female sex, greater frailty, comorbidities, previous psychiatric illness), the nature of the acute illness (e.g., sepsis, cardiac arrest, acute respiratory distress syndrome, or multiorgan failure), delirium, and treatments in the ICU (e.g., longer time on invasive mechanical ventilation and use of vasoactive agents, corticosteroids, and neuromuscular blocking agents), although findings conflict across studies [13, 15, 26]. PICS requires long-term and often multidisciplinary healthcare, resulting in substantial costs. Moreover, family members, notably those acting as informal caregivers, may be affected also, the condition then being termed PICS-family. Thus, PICS is now recognised as a public health issue [27–29]. At a Society of Critical Care Medicine meeting held in 2012, PICS was identified as deserving priority research and management status, with the aim of improving long-term outcomes of ICU survivors [17, 30].

PICS symptoms can be screened via a specific in-person evaluation led by an intensivist, who uses validated instruments and tests as screening tools [31, 32]. If PICS symptoms are diagnosed, the intensivist determines the specific needs of the patient and coordinates the provision of appropriate care by the general practitioner and, if needed, other specialists. In response to the large increase in the number of ICU survivors that followed the initial COVID-19 waves, the French central health authority (*Haute Autorité de Santé*, HAS) developed guidelines on PICS screening, which were published in June 2023 [33]. The recommendations include a post-ICU visit conducted by an intensivist in patients at risk for PICS and a list of appropriate evaluation tools. However, the availability and conduct of these visits in France is not well known.

We therefore conducted a survey of multiple French ICUs to evaluate current practice regarding the availability of post-ICU visits and the evaluation methods used to detect PICS. The data will provide a background against which the impact of the 2023 HAS guidelines can be evaluated.

## Methods

### Study design

We conducted an online survey by sending a 43-item questionnaire to the French ICUs identified in previous nationwide surveys as accredited for *Médecine Intensive Réanimation* (MIR) for training intensivists and practicing intensive care in adults [34, 35]. In France, MIR-accredited ICUs provide either medical or both medical and surgical care; thus, surgery-only ICUs were not invited to participate.

For each ICU, any of three investigators (MA, MC, or EC) e-mailed the survey to the intensivist in charge of post-ICU visits if available and to the ICU head otherwise. Thus, each ICU was sent a single survey. Non-respondents received two reminders at two-week intervals, then either a third e-mail reminder or a phone call. All e-mail and phone calls occurred between 13 February 2023 and 23 June 2023, that is, immediately before the publication of the French HAS guidelines.

The survey complied with regulations on research not involving humans (MR-004 reference method) established by the French data protection authority (*Commission Nationale de l’Informatique et des Libertés*, CNIL).

### Survey

The survey (Supplementary File 1) was created using Google Forms (Google, Mountain View, CA). The items were selected by two investigators (MA and EC). Next, six intensivists (GB, LD, JBL, MM, AS, and JL) with experience in post-ICU visits reviewed the survey, which was modified according to their comments. The final version was validated by MA and EC.

The 43-item survey covered the following domains: general information about the ICU (type of hospital, number of beds, number of full-time intensivist equivalents, and number of admissions per year); reasons for not offering post-ICU visits, if such was the case, and plans to start offering such visits within the next year; and, for ICUs with visits, organisation of the visits, criteria for patient eligibility to a visit, evaluations done during the visits (instruments and physical tests used, laboratory tests, and imaging studies), and healthcare pathways open to patients after the visit, notably whether the ICU worked with an established network of specialists experienced in managing PICS. The most common response option was a choice among multiple possibilities, including “other” with a space for providing free-form text details; for some questions, the response was dichotomous (yes or no) or given as free-form text.

### Primary and secondary objectives

The primary objective was to determine the proportion of participating ICUs offering post-ICU outpatient visits. The secondary objectives were to describe the type of ICUs offering such visits, the nature of the evaluations performed during the visits, and the reasons for not offering visits.

### Statistical analysis

The Google Form data were extracted into a Microsoft Excel spreadsheet (version 2016, Microsoft Corporation, Redmond, WA). Qualitative variables were described as n (%) and quantitative variables as median [interquartile range]. Comparisons were made with Fisher’s exact test for qualitative variables and Student’s *t* test for quantitative variables. As for the nature of the dataset (survey), we did not expect much missing data and thus did not use any imputation method. Values of *p* smaller than 0.05 were considered significant. All statistical analyses were performed using the “R statistics programme”, version 3.5.0 (R Foundation for Statistical Computing, Vienna, Austria; www.r-project.org).

## Results

**Fig. 1:**
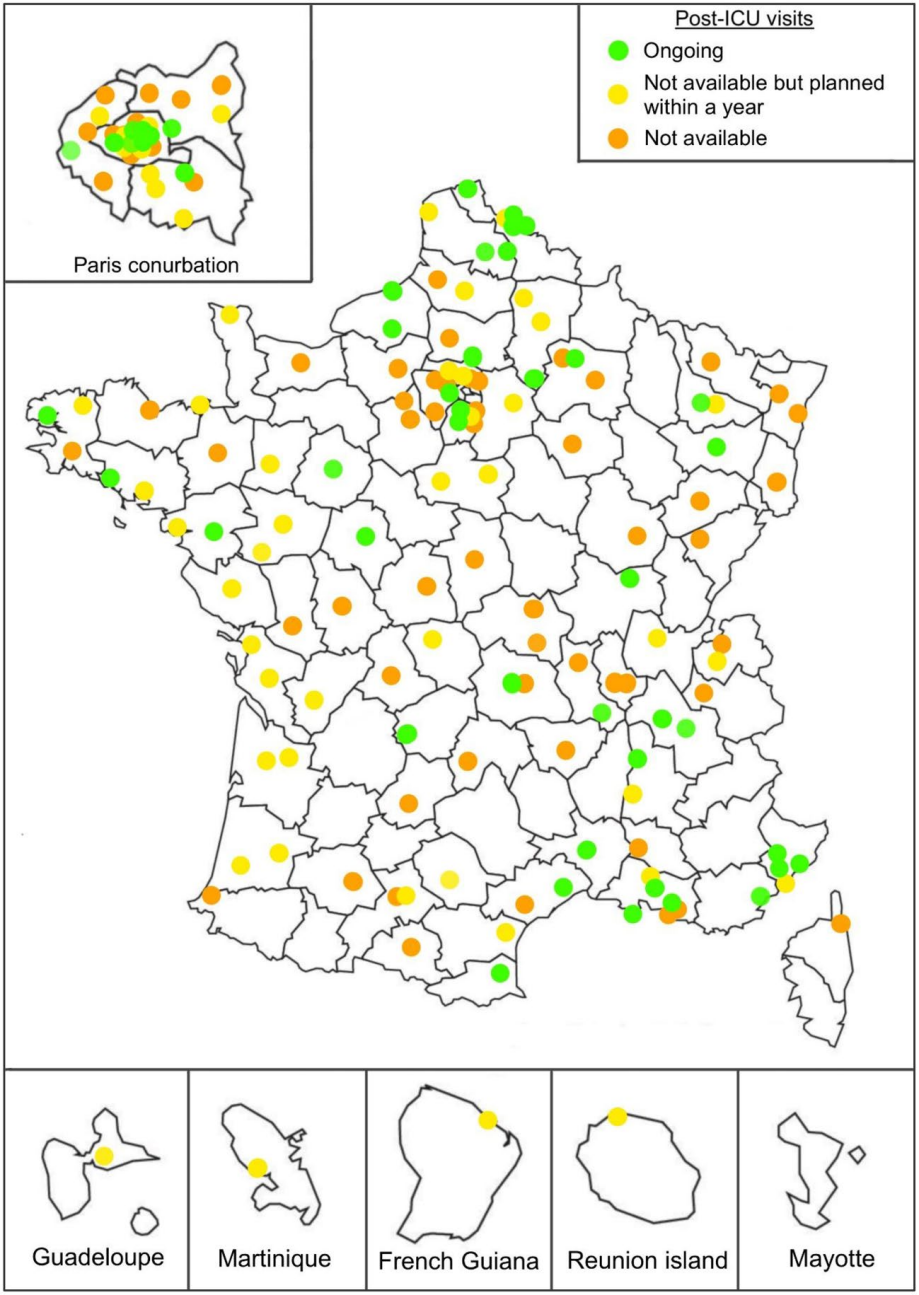
Distribution of the 161 surveyed intensive care units (ICUs) in mainland and overseas France

Among the 252 French ICUs surveyed, 161 (63.9%) responded. Figure 1 shows that the respondent ICUs were evenly distributed across the mainland and overseas regions of France.

### Characteristics of the respondent ICUs

Of the 161 respondent ICUs, 98 (61%) were in community hospitals, 47 (29%) in university hospitals, and 16 (10%) in private hospitals. The median number of ICU beds was 18 [15–27] and the median number of full-time intensivist equivalents was 7 [5.6–9.0]. The median annual number of ICU admissions was 900 [700–1110].

Post-ICU outpatient visits were offered by 46/161 (28.6%) ICUs. ICUs with visits had significantly more beds (21 [15–24] vs. 18 [15–24], *p* = 0.02). University-hospital ICUs had more often visits than ICUs in other hospital types, although the difference was not significant (43% vs. 23%, *p* = 0.058). Neither the number of annual admissions nor the number of full-time intensivist equivalents differed significantly between ICUs with vs. without post-ICU outpatient visits.

### Organisation of post-ICU visits (46 ICUs)

Table 1 provides details on the organisation of post-ICU visits. The most common scenario was a single in-person visit 3 to 6 months after ICU discharge. In all cases, an intensivist is present, as well as nurses in 16 (35%) ICUs and psychologists in 13 (28%) ICUs. Most ICUs with post-ICU visits provided such visits once a week, on a set day (*n* = 33, 72%). The most common visit duration was 30–60 min (*n* = 20). The median number of patients who attended post-ICU visits was 50 [30–100] per ICU and per year, accounting for about 5% of annual ICU admissions.

**Table 1:** Organisational features of post-ICU outpatient visits (46 intensive care units)

Seven survey items assessing visit organisation	Median [IQR] or *n* (%)
Estimated number of patients seen annually	50 [30–100]
*How many visit(s) do you schedule per patient?* One Two More than two As needed	29 (63) 4 (9) 4 (9) 9 (20)
*How long after ICU discharge is/are the visit(s) scheduled?* ^*a*^ 1 month 3 months 6 months 12 months 15 months	6 (13) 25 (54) 20 (43) 6 (13) 1 (2)
*How do the visits take place?* ^*a*^ Face-to-face visit Day-hospital admission Remote visit	31 (67) 14 (30) 3 (6)
*How long does each visit last?* 30 min to 1 h >1 h to 2 h Half a day A day	34 (74) 6 (13) 3 (7) 3 (7)
*How frequently does your ICU provide post-ICU visits?* Several times a week Once a week Every two weeks Every month No set interval	4 (9) 27 (59) 7 (15) 6 (13) 2 (4)
*What kind of healthcare professionals are present at the visit?* ^*a*^ Intensivist/ anaesthesiologist Physician in another specialty Nurse Psychologist Physical therapist Nutritionist Nursing assistant Speech therapist Occupational therapist Clinical research associate	46 (100) 9 (20) 16 (35) 13 (28) 8 (17) 6 (13) 2 (4) 1 (2) 1 (2) 1 (2)
*Is the physician leading the visit* An ICU physician specifically in charge of post-ICU visits? Any ICU physician?	35 (76) 11 (24)
*What is the critical-care experience of the physician leading the visit?* Senior with < 5 years of experience Senior with ≥ 5 years of experience Junior	22 (48) 22 (48) 2 (4)
*Where do the visits take place?* Dedicated out-patient visit room outside the ICU Dedicated out-patient visit room in the ICU Room in the ICU, not used only for outpatient visits Intensivist’s office	32 (70) 8 (17) 4 (9) 2 (4)

*Abbreviation* ICU: intensive care unit

^a^More than one answer was possible for each respondent ICU

Supplementary File 2 shows that the main eligibility criteria for post-ICU visits were ICU stay and/or having required invasive mechanical ventilation for at least 48 h or having experienced cardiac arrest, septic shock, or acute respiratory distress syndrome.

### Evaluations done during the post-ICU visit

Table S1 in the Supplementary File lists the evaluations performed during the post-ICU visits. A thorough physical examination and validated psychometric instruments were used in 56.5% and 80% of ICUs, respectively. ICU-acquired weakness and dyspnoea were commonly evaluated using the 6-minute walk test, modified Medical Research Council scale, and New York Heart Association scale. Nearly three-fourths of ICUs included an assessment of anxiety and depression, usually with the Hospital Anxiety and Depression Scale; two-thirds tested for PTSD symptoms using the Impact of Events Scale or the PTSD checklist for DSM-5; two-thirds assessed quality of life, generally with the 36-item Short Form Health Survey; and half tested for cognitive dysfunction, usually with the Montreal Cognitive Assessment or Mini-Mental State Examination. In contrast, phonation and swallowing were not usually investigated. Almost all ICUs evaluated functional disabilities and autonomy, using various scales, notably the Modified Rankin Scale, Katz Index of Independence in Activities of Daily Living, Instrumental Activities of Daily Living and Barthel Index.

In most ICUs, the place of residence, return to work, and need for help from carers and/or technical aids were recorded. Income and return to recreational activities were rarely documented. About a third of ICUs delivered information on advance directives and treatment limitations. Only 3/46 ICUs discussed organ donation. Information on the ICU stay was given by only a minority of ICUs, with 7/46 returning a patient diary and 4/46 giving a comprehensive summary of the stay. Few ICUs performed investigations, which usually consisted in blood tests (13/46). Nine ICUs reported specifically assessing relatives, including five with specific PICS-family screening.

### Healthcare network for PICS management

Twenty-five out of the 46 ICUs (54%) with post-ICU follow-up worked with an established network of healthcare professionals experienced in managing PICS. The professionals involved were pulmonologists (*n* = 17, 68%), cardiologists (*n* = 16, 64%), nephrologists (*n* = 11, 44%), psychiatrists (*n* = 10, 40%), neurologists (*n* = 10, 40%), rehabilitation physicians (*n* = 9, 36%), ear/nose/throat specialists (*n* = 8, 32%), physiotherapists (*n* = 10, 40%), and psychologists (*n* = 9, 36%). The reasons for not having such a network were chiefly organisational (*n* = 13), although in some cases either no healthcare professionals were interested in PICS (*n* = 5) or none had training in PICS management (*n* = 3). A network was in the process of being established in eight ICUs.

### ICUs without post-ICU visits

**Fig. 2:**
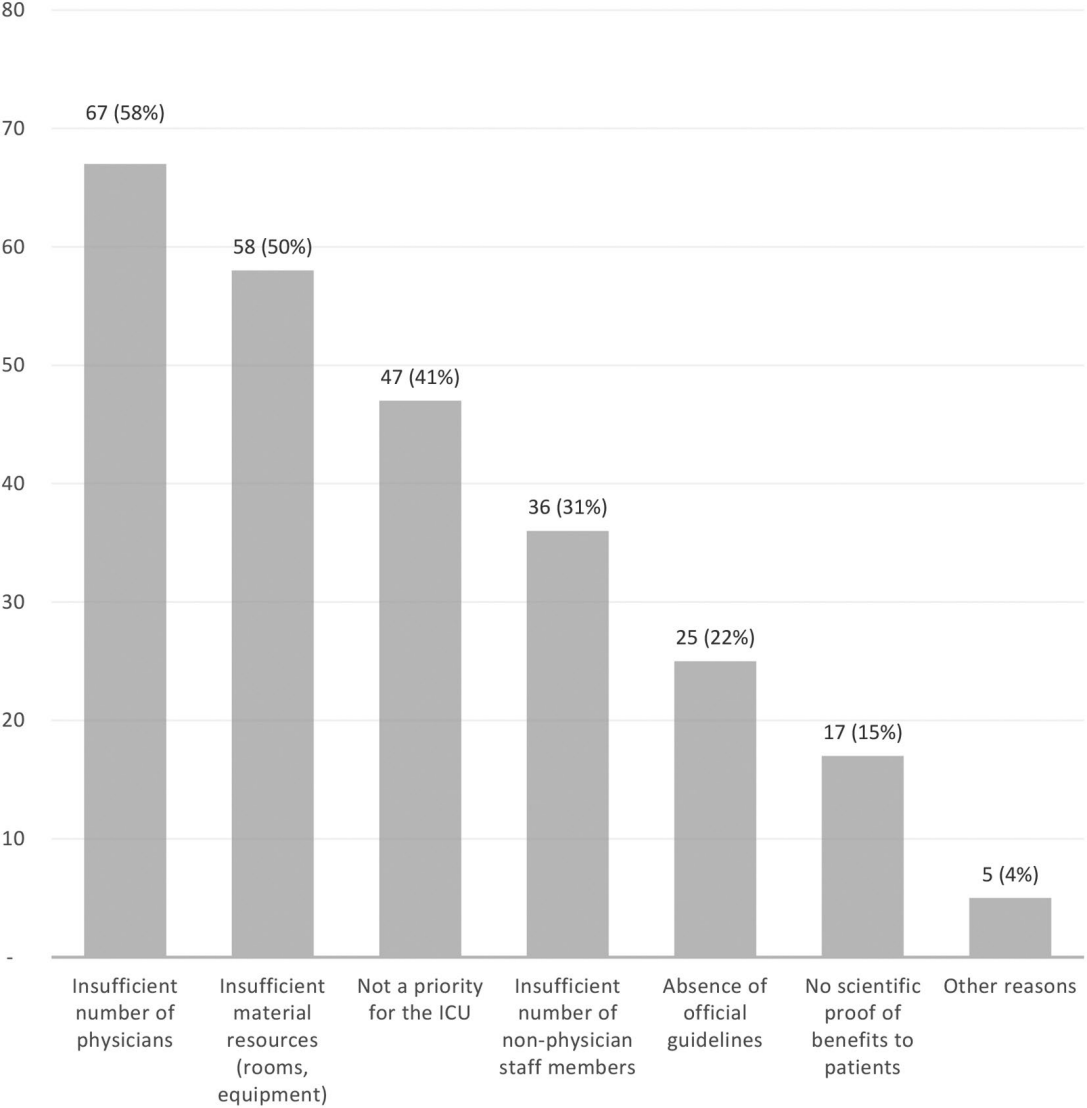
Reasons for not offering visits after intensive-care-unit (ICU) discharge (*n* = 115). The data are absolute values. Each respondent ICU could provide more than one answer

Of the 161 ICUs, 115 (71.4%) did not offer post-ICU visits. Figure 2 shows the main reasons. Insufficient staff and equipment was a common obstacle, and 40.9% of these ICUs felt follow-up was not a priority. One ICU reported that post-ICU visits had been started but not continued. Importantly, 50/115 (43.5%) ICUs reported having plans to start follow-up visits within the next year.

## Discussion

### Key findings

Our survey, with a high response rate of 64%, provides a comprehensive overview of post-ICU visits of French medical and medico-surgical ICUs. Visits were available in 28.6% of ICUs overall and were more commonly offered by larger ICUs and by ICUs in university hospitals. In most cases, a single, intensivist-led visit was scheduled, 3 to 6 months after ICU discharge. Screening for PICS involved validated instruments in 80% of ICUs. The annual number of visits was about 50, accounting for about 5% of admissions. About half the ICUs with visits worked with established networks of professionals providing care to patients with PICS and a further 17% were setting up such a network. Of the 115 ICUs without post-ICU visits, 50 (43%) had plans to start visits within the next year. Finally, insufficient training or interest in PICS and lack of staff and equipment were common obstacles to post-ICU follow-up and care.

### Comparison with previous studies

Although awareness of PICS is increasing among intensivists and guidelines have been issued [33, 36], studies have often been confined to specific acute illnesses (e.g., cardiac arrest, acute respiratory distress syndrome, or septic shock). High prevalences of long-term post-ICU symptoms have been documented [37–39]. Nonetheless, screening for PICS is not consistently available, and the battery of evaluation tools necessary to detect PICS has not been standardised. Previous surveys found that the proportion of ICUs offering follow-up visits ranged from 1.9% in Australia in 2020 to 73.9% in the UK in 2021 [40–42]. The high proportion in the UK followed the issuance of guidelines in 2009, which were, however, slow to take effect, as the proportion in 2013 was still only 27.3% [43]. This last value was similar to that in our study done just before guidelines were issued in France [33]. The fact that 43.5% of ICUs without visits have plans to start visits within the next year is encouraging.

Reported post-ICU follow-up modalities vary substantially. A Scandinavian study identified four models depending on whether visits were led by nurses or multidisciplinary teams and on whether a patient diary was created [42]. Nurse-led visits with a patient diary and a focus on understanding the ICU experience was the most common approach. In the UK, the main modality was a single in-person visit 2 to 3 months after discharge, with assessments by multiple professionals including a nurse, an intensivist, and a physiotherapist [41]. In nearly half the cases, no psychological assessment was performed. In our study, a single visit led by an intensivist and performed 3 to 6 months after discharge was the most common set-up. A psychologist was involved in only 28% of ICUs. Understanding the ICU experience was not usually the focus of the visits, with only 15% of ICUs returning a patient diary at follow-up. Instead, the main goal was to detect PICS symptoms. In three quarters of ICUs, an intensivist was specifically in charge of the post-ICU visits, as recommended by the HAS [33]. However, the full spectrum of instruments recommended to assess the physical, psychological, and cognitive spheres, as well as self-sufficiency and quality of life, was rarely used.

In our survey, the patient selection criteria used by the ICUs with visits were among the many reported risk factors for PICS, namely, prolonged ICU stay and/or invasive mechanical ventilation, acute respiratory distress syndrome, septic shock, and cardiac arrest [1, 2, 13, 26, 40, 41, 43, 44]. However, other risk factors such as older age, clinical frailty, delirium, and impaired functional status were not reported as selection criteria. Only 5% of admissions were followed by an offer to attend a post-ICU visit. These data suggest that French intensivists may need to broaden their criteria for post-ICU follow-up. However, the extent to which resource limitations led to the application of restrictive criteria was not assessed in our survey. Also, staffing shortages may have contributed to limit the range of specialists involved in post-ICU visits.

The barriers to offering post-ICU follow-up were investigated in the nationwide Australian survey [40]. Of the 107 respondent ICUs, only two offered visits. Lack of funding was the most common barrier. Other obstacles were absence of epidemiological data on PICS outcomes in Australia and lack of proof that post-ICU visits improved those outcomes. In our survey, lack of proof of efficacy also led some ICUs to deem that post-ICU visits were not a priority. A paucity of human and material resources was also reported. Further research is needed to assess whether post-ICU visits, followed by the appropriate management of any detected PICS manifestations, improves patient outcomes.

### Study implications

Of the 161 ICUs that responded to our survey, 28.6% offered post-ICU visits and only a minority of ICU patients were offered follow-up visits. Notably, a further 31% ICUs intended to offer visits within the next year. French intensivists can now rely on the guidelines issued recently by the French central health authority (HAS) to provide direction on the diagnosis and management of PICS. In addition to these official guidelines and in light of our findings, we provide exploratory suggestions for teams that would want to start or strengthen their post-ICU follow up and in order to unify practices. We showed that patient selection criteria as it is, seems too narrow, allowing to assess only a small sample of ICU survivors. ICU teams should consider broadening their criteria based on recognized PICS risk factors in order to be able to assess a wider range of patient. Also, the development of a minimum standardised dataset to be collected by all ICUs could be valuable. Our findings suggest that the evaluations of physical, mental, and cognitive health could include the WHO Performance Status, Frailty Scale, Hospital Anxiety and Depression scale, Impact of Events Scale-revised, Short Form 36, Montreal Cognitive Assessment, 6-minute walk test, and modified Rankin Scale. These simple and freely available tools require little time and no specific training.

Moreover, intensivists might anticipate the possible need for referrals after screening (e.g., to the primary-care physician, usual specialists, or specialists identified by the ICU team) to ensure that all patient needs are met while also improving patient satisfaction [45].

Finally, nationwide, a network coordinated by critical-care societies and designed to collect data on PICS from ICUs providing post-ICU follow-up would help to unify practices, produce additional evidence on PICS, evaluate the impact of follow-up on patient outcomes, and encourage funding of this activity.

### Strengths and limitations

The survey design with the collection of data based on reports by ICUs is a limitation of our work. However, the survey was sent either to the intensivist in charge of post-ICU visits or to the ICU head, who would be expected to provide reliable answers. Second, our survey included medical and medical-surgical ICUs and its findings therefore do not reflect post-ICU follow-up provided by surgery-only ICUs. Nonetheless, medical and medical-surgical ICUs account for 80% of all ICUs in France. [46] Third, our study focused on ICUs for adult patients, in consequence, our data are not representative of paediatric ICUs. Fourth, slightly over a third of the invited ICUs did not respond to the survey. A reasonable assumption is that non-respondents were more likely to have no post-ICU follow-up programme. Thus, the proportion of ICUs offering such visits may have been overestimated in our study. Moreover, the survey was not built using a standard method, such as Delphi rounds. Instead, the items were chosen by intensivists who had experience with post-ICU visits. Our study also has strengths. It provides the first comprehensive picture of post-ICU follow up throughout French medical ICUs. The high response rate indicates strong external validity. The survey was detailed, providing information on the availability of visits, the evaluations performed, plans to offer visits in the future, and obstacles to such plans.

## Conclusion

The 28.6% proportion of ICUs with follow-up visits is too low, but if plans to set up such visits are successful, within a year, this proportion may increase to about three-fifths. The 5% proportion of patients admitted in ICU annually, attending post-ICU follow up is insufficient. The issuance of national guidelines just after our survey can be expected to further increase the proportion of ICUs providing post-ICU visits, while also improving the quality of the evaluations offered. Efforts are needed to raise awareness among healthcare policy makers and hospital administrators that funding for post-ICU follow-up and subsequent care is needed. The data collected during post-ICU visits will allow further investigations of PICS epidemiology, manifestations, and management.

### Electronic supplementary material

Below is the link to the electronic supplementary material.

Supplementary Material 1

## Data Availability

The datasets used and/or analysed during the current study are available from the corresponding author on reasonable request.

## Abbreviations

HAS: Haute Autorité de Santé (French health and drugs agency)
ICU: Intensive care unit
PICS: Post-intensive care syndrome
PTSD: Post-traumatic stress disorder
