# Supplementary material for: Post-intensive care syndrome screening: a French multicentre survey
Source: Ann Intensive Care. 2024 Jul 9;14:109. doi: 10.1186/s13613-024-01341-y (PMC11233491; doi:10.1186/s13613-024-01341-y)
Supplement: Supplementary file 1 — Supplementary Material 1 [file 13613_2024_1341_MOESM1_ESM.docx]

**SUPPLEMENTARY MATERIAL**

**TABLE OF CONTENTS**

Supplementary File 1: the online survey questionnaire ……………………………………….p 2

Supplementary File 2. Criteria used to define patients eligible for post-ICU outpatient

follow-up (46 intensive care units)…………………………………………………………….p 19

Supplementary Table S1: Evaluations performed during the post-ICU follow-up

visits (46 intensive care units)…………………………………………………………………p 21

## Supplementary File 1: The online questionnaire

**Survey on Current Practices for ICU Survivor Follow-up by ICUs in France**

*Items marked with an asterisk must be answered.

1. E-mail address^*^
2. What city is your ICU located in? ^*^
3. What type of hospital is your ICU located in? ^*^

*Please select a single reply.*

University hospital

Regional hospital

Community hospital

Private hospital

1. What is the number of beds in your ICU and step-down unit (report the total)?
2. How many intensivists (full-time equivalents) does your ICU have?
3. How many admissions are there each year to your ICU and step-down unit (report the total)?
4. Does your ICU offer survivors one or more post-ICU visits?^*^

*Please select a single reply.*

Yes *Please skip to item 10*

Non *Please continue to the next item (8).*

1. Your ICU does not offer post-ICU visits. Please select one or more of the reasons listed below.

Inadequate number of physicians

Inadequate number of nurses

Inadequate material resources (dedicated room, equipment)

Not currently viewed as a priority in this ICU

Absence of scientific evidence that post-ICU visits improve patient outcomes

Absence of official recommendations about post-ICU visits

Other (please specify):

1. Your ICU does not offer post-ICU visits: are there any plans to offer post-ICU visits within the next year?

*Please select a single reply.*

Yes

No

**Post-ICU VISIT MODALITIES AND ORGANISATION**

Which patients are offered a post-ICU visit?

*Please select one or more of the categories below.*

All patients discharged alive from the ICU

Patients with ICU or step-down unit stays ≥24 h

Patients with ICU or step-down unit stays ≥48 h

Patients with ICU or step-down unit stays ≥72 h

Patients given invasive mechanical ventilation for ≥24 h

Patients given invasive mechanical ventilation for ≥48 h

Patients given invasive mechanical ventilation for ≥72 h

Patients who received renal replacement therapy during the stay

Patients who received vasoactive agents in any dose

Patients who received vasoactive agents in a dose above a cut-off

Patients who experienced cardiac arrest (as the reason for admission and/or during the stay)

Patients who experienced sepsis or septic shock (as the reason for admission and/or during the stay)

Patients who experienced acute respiratory distress syndrome (as the reason for admission and/or during the stay)

Patients included in a clinical research study

Other (please specify):

1. How many patients attend post-ICU visits each year?
2. Is each patient scheduled for a single visit or for several visits?

*Please select one or more of the options below.*

A single visit

Two visits

More than two visits

Other (please specify):

1. What is the time from ICU discharge to the scheduled post-ICU visit or visits?

*Please select one or, in the event of multiple visits, several of the options below.*

1 month

3 months

6 months

12 months

15 months

18 months

24 months

Other (please specify):

1. What are the modalities of the post-ICU visits?

*Please select one or more options below.*

In-person visit with an intensivist

In-person multidisciplinary visit

In-person visit with an intensivist by telephone or video link (teleconsultation)

Multidisciplinary visit by telephone or video link (teleconsultation)

Day hospital

Other (please specify):

1. Who performs the post-ICU visits?

*Please select one or more options below.*

Intensivist or anaesthesiologist

Physician in another specialty (e.g., rehabilitative medicine)

Nurse

Physical therapist

Psychologist

Neuropsychologist

Nutritionist

Speech therapist

Occupational therapist

Other (please specify):

1. Among physicians working in the ICU, which ones perform post-ICU visits?

*Please select one or more options below.*

Any of the ICU physicians

One or more ICU physicians specifically in charge of post-ICU visits

Other (please specify):

1. What is the experience level of the ICU physicians who perform post-ICU visits?

*Please select one or more options below.*

Resident or junior intensivist/anaesthesiologist

Senior intensivist/anaesthesiologist with 1–2 years’ experience

Senior intensivist/anaesthesiologist with 3–5 years’ experience

Senior intensivist/anaesthesiologist with >5 years’ experience

Other (please specify):

1. Where do the post-ICU visits take place?

*Please select one or more options below.*

Dedicated room in the ICU

Dedicated room outside the ICU

ICU physician office, made available for the duration of the post-ICU visit

Room in the ICU, made available for the duration of the post-ICU visit

Other (please specify):

1. How much time is set aside for post-ICU visits?

*Please select one or more options below.*

One half day per week

One day per week

One half day every two weeks

One half day every two weeks

One half day per month

One day per month

Other (please specify):

1. Is the time slot set aside for post-ICU visits fixed or variable?

*Please select a single reply.*

Always on the same day

The day varies depending on availability (room, physicians…)

Other (please specify):

1. How long does a post-ICU visit last?

*Please select one or more options below.*

30 minutes

1 hour

2 hours

Half a day

Other (please specify):

*Passer à la question 22*

Clinical and psychometric tests used during post-ICU visits

1. Does the post-ICU physical examination routinely assess all systems or focus on systems identified by patient complaints?

*Please select a single reply.*

All systems

Only systems identified by complaints

1. Which of the following clinical variables are recorded routinely during each post-ICU visit?

*Please select one or more options below.*

Blood pressure

Heart rate

Breathing rate

Pulse oximeter reading

Body temperature

Body weight

Pain score on a visual analogue scale or verbal rating scale

None of the above

Other (please specify):

1. Physical and psychometric assessments: do you use one or more specific instruments during the post-ICU visit?

*Please select a single reply.*

Yes

No

*Please skip to item 34 if the answer was “No”.*

**MULTIMODAL EVALUATION USING TESTS AND SCORES**

1. Evaluation of general health and frailty

Which of the following instruments for evaluating general health and frailty are used during the post-ICU visits?

*Please select one or more options below.*

WHO Performance Status

Karnofsky Performance Scale

Clinical Frailty Scale

None

Other (please specify):

1. Mental-health evaluation

Which of the following instruments for evaluating depression and anxiety are used during the post-ICU visits?

*Please select one or more options below.*

Hamilton Depression Rating Scale (HDRS or HAMD)

Hospital Anxiety and Depression Scale (HADS)

Montgomery and Asberg Depression Rating Scale (MADRS)

Patient Health Questionnaire depression scale (PHQ-8)

Generalised Anxiety Disorder (GAD-7)

None

Other (please specify):

1. Which of the following instruments for evaluating post-traumatic stress disorder (PTSD) are used during the post-ICU visits?

*Please select one or more options below.*

PTSD Check List for DSM-5 (PCL 5)

Clinical Administered PTSD Scale for DSM-5 (CAPS 5)

Impact of Events Scale (IES)

None

Other (please specify):

1. Which of the following instruments for evaluating quality of life are used during the post-ICU visits?

*Please select one or more options below.*

Short-Form Health Survey (SF-36)

EuroQol-5D-5L

None

Other (please specify):

1. Which of the following instruments for evaluating cognitive function are used during the post-ICU visits?

*Please select one or more options below.*

Mini Mental State Examination (MMSE)

Montreal Cognitive Assessment (MoCA)

Frontal Assessment Battery at bedside

Five-word test

Grober-Buschke test (verbal episodic memory)

Galveston Orientation and Amnesia Test (GOAT)

Repeatable Battery for the Assessment of Neuropsychological Status (RBANS)

Trail-Making Test (TMT) A and B

Digit Span Memory Test

None

Other (please specify):

1. Is cognitive function assessed by a physician or by a neuropsychologist?

*Please select a single reply.*

Physician

Neuropsychologist: screening tests

Neuropsychologist: extensive neuropsychological evaluation

Other (please specify):

1. Which of the following instruments for evaluating physical and functional status are used during the post-ICU visits?

*Please select one or more options below.*

New York Heart Association (NYHA) classification of dyspnoea

Modified Medical Research Council (mMRC) Dyspnoea Scale

Handgrip strength measured using a dynamometer

Short Physical Performance Battery (SPPB)

Physical Function ICU Test (PFIT)

Borg Rating of Perceived Exertion

Berg Balance Scale

Six-Minute Walk Test

Timed Up-and-Go test

One-Minute Sit-and-Stand Test

None

Other (please specify):

1. Which of the following instruments for evaluating swallowing and phonation are used during the post-ICU visits?

*Please select one or more options below.*

Eating Assessment Tool (EAT-10)

Volume-Viscosity Swallow Test

Grade Roughness, Breathiness, Asthenia, STrain (GRBAS)

None

Other (please specify):

1. Which of the following instruments for evaluating functional status, autonomy, and disability are used during the post-ICU visits?

*Please select one or more options below.*

Katz Activities of Daily Living Scale

Lawton-Brody Instrumental Activities of Daily Living Scale

Barthel Index

*Mesure d'Indépendance Fonctionnelle* (MIF) (functional self-sufficiency measure)

Functional Activities Questionnaire (FAQ)

Modified Rankin Scale

Glasgow Outcome Scale

None

Other (please specify):

1. In patients enrolled in one or more clinical research studies during the ICU stay, is/are the study-related follow-up visit(s) and the post-ICU visit(s) conducted jointly?

*Please select a single reply.*

Yes, always

Yes, sometimes

Never

Other (please specify):

1. Evaluation of the relatives: Does the post-ICU visit include a specific evaluation of the relatives?

*Please select a single reply.*

Yes: PICS-family assessment

Yes: other assessment

No: relatives may be present but are not specifically evaluated

No: no relatives attend the visit

Other (please specify):

1. Which of the following data on the patient’s living conditions are recorded during the post-ICU visit?

*Please select one or more options below.*

Place of residence

Return to work

Income

Assistance from professional carers

Technical aids

None

Other (please specify):

1. Among the following items of information, which are given to the patient during the post-ICU visit?

*Please select one or more options below.*

Information on advance directives

Information on organ donation

Delivery of the patient diary if applicable

Discussion of potential treatment limitations and/or criteria for repeat ICU admission

None

Other (please specify):

**INVESTIGATIONS**

^38.^ Among the following laboratory tests, which are performed ROUTINELY for post-ICU visits (either shortly before the visit or on the day of the visit)?

*Please select one or more options below.*

Complete blood count

Serum electrolytes, urea, and creatinine

Liver function tests

Prothrombin time (PT), partial thromboplastin time (aPTT)

Arterial blood gas test

C-reactive protein (CRP)

Fasting blood glucose

Glycated haemoglobin

Serum albumin

Pre-albumin

Thyroid-stimulating hormone

Serum cortisol level

Blood lipid profile

Proteinuria on a single sample or 24-h collection

None

Other (please specify):

1. Among the following imaging studies and functional tests, which are performed ROUTINELY for post-ICU visits (either shortly before the visit or on the day of the visit)?

*Please select one or more options below.*

Electrocardiogram

Chest radiograph

Computed tomography of the chest

Lung function testing

Transthoracic echocardiogram

Electroneuromyography

None

Other (please specify):

**FOLLOW-UP AFTER THE POST-ICU VISIT**

1. Who is the post-ICU visit report sent to?

*Please select one or more options below.*

Patient

Primary-care physician

Specialists providing follow-up to the patient

None

Other (please specify):

1. Does your ICU work with established healthcare networks to meet specialist-care needs identified by the post-ICU visit?

*Please select a single reply.*

Yes *Please continue from item 42.*

No *Please skip to item 45.*

**POST-ICU HEALTHCARE NETWORK**

1. Does the post-ICU healthcare network to which you direct patients consist of:

*Please select one or more options below.*

A specialised network involving physicians working in publicly funded hospitals

A specialised network involving physicians working in privately funded hospitals

A specialised network involving community physicians

Other (please specify):

1. Which types of healthcare professionals are involved in the post-ICU healthcare network?

*Please select one or more options below.*

Physical therapist

Adapted physical activity professional

Rehabilitation medicine professional

Pulmonologist

Nephrologist

Gastroenterologist/hepatologist

Neurologist

Cardiologist

Rheumatologist

Psychiatrist

Oto-rhino-laryngologist

Clinical psychologist

Neuropsychologist

Speech therapist

Occupational therapist

Nutritionist

Other (please specify):

1. How much training/experience in post-ICU health issues do these professionals have?

*Please select one or more options below.*

Theoretical training on post-ICU health issues

Clinical experience, no specific training

Other (please specify):

1. Your ICU does not have an established healthcare network for patients with post-ICU syndrome: Why?

*Please select one or more options below.*

Not currently identified as a priority

No specialised physicians are interested in post-ICU syndrome

No physicians have been trained in managing post-ICU syndrome

Organisational challenges

A network is currently being set up

Other (please specify):

Supplementary File 2. Criteria used to define patients eligible for post-ICU outpatient follow-up

Sepsis or septic shock^d^

Acute respiratory distress syndrome^d^

Inclusion in a research protocol

Patients with specific conditions^e^

PTSD risk factors

Other reasons^f^

None (all patients eligible)

ICU length of stay^a^

Mechanical ventilation^b^

Renal replacement therapy

Vasopressor support^c^

Cardiac arrest^d^

The data are absolute values. Each respondent ICU could provide more than one answer.

^a^≥48 h, n=6; ≥72 h, n=6; ≥7 days, n=8

^b^≥24 h, n=6; ≥48 h, n=11; ≥72 h, n=8

^c^any dose, n=8; dose > a cut-off, n=2

^d^reason for ICU admission or development during the ICU stay

^e^Covid-19, n=2; chronic respiratory failure, n=2; brain damage, n=1; acute kidney failure KDIGO 3 n=1; body mass index <18 or >35 kg/m², n=1

^f^Physician’s judgment, n=2; hyperbaric oxygen therapy, n=1; ethical visit, n=1

ICU: intensive care unit; KDIGO 2: Kidney Disease: Improving Global Outcomes stage 2; PTSD: post-traumatic stress syndrome

## Supplementary Table S1: Evaluations performed during the post-ICU follow-up visits (46 intensive care units)

| **Nine questionnaire items assessing visit organisation** | **n (%)** |
| --- | --- |
| Which **general health** **variables** do you record routinely during the visit?  *Blood pressure*  *Heart rate*  *Respiratory rate*  *SpO_2_*  *Body temperature*  *Body weight*  *Pain intensity using a visual analogue scale*  *None* | 34 (74)  33 (73)  23 (50)  33 (73)  13 (28)  31 (67)  16 (35)  6 (13) |
| Among the instruments listed below, which do you use to assess **general health and frailty?**  *WHO Performance Status*  *Frailty scale*  *Karnofsky Performance Scale* | 11 (24)  11 (24)  4 (9) |
| Among the instruments listed below, which do you use to assess **depression and anxiety**?  *Hospital Anxiety and Depression Scale*  *Personal Health Questionnaire Depression Scale, 8 items*  *Generalised Anxiety Disorder, 7 items*  *Other^a^* | 28 (60)  3 (6)  1 (2)  2 (4) |
| Among the instruments listed below, which do you use to assess **post-traumatic stress disorder**?  *Impact of Events Scale*  *PTSD checklist for DSM-5*  *Other^b^* | 16 (35)  11 (24)  2 (4) |
| Among the instruments listed below, which do you use to assess **quality of life**?  *Short Form-36*  *EuroQol-5D-5L*  *Other^c^* | 20 (44)  6 (13)  3 (7) |
| Among the instruments listed below, which do you use to assess **cognitive function**?  *Montreal Cognitive Assessment*  *Mini Mental State Examination*  *Five-Word Test*  *Frontal Assessment Battery at bedside*  *Galveston Orientation and Amnesia Test* | 12 (26)  5 (11)  3 (7)  1 (2)  1 (2) |
| Among the instruments listed below, which do you use to assess **physical and functional status**?  *Six-minute walk test*  *New York Heart Association Functional Status*  *Modified Medical Research Council dyspnea scale*  *Handgrip strength with handheld dynamometer*  *Borg Rating of Perceived Exertion*  *1-minute Sit-to-Stand Test*  *Physical Function in ICU Test*  *Timed Up and Go Test* | 16 (35)  14 (30)  9 (20)  6 (13)  3 (7)  3 (7)  1 (2)  1 (2) |
| Among the instruments listed below, which do you use to assess **phonation and swallowing**?  *Eating Assessment Tool-10*  *Volume-viscosity swallow test* | 1 (2)  1 (2) |
| Among the instruments listed below, which do you use to assess **functional abilities, autonomy, and disability**?  *Modified Rankin Scale*  *Katz Index of Independence in Activities of Daily Living*  *Instrumental Activities of Daily Living*  *Barthel Index*  *Functional Activities Questionnaire*  *Glasgow Outcome Scale*  *Other^d^* | 10 (22)  9 (20)  8 (17)  8 (17)  5 (11)  5 (11)  1 (2) |

More than one answer was possible for each respondent ICU.

^a^Beck Depression Inventory, n=1; 4-item Personal Health Questionnaire Depression Scale, n=1

^b^PTSD Symptom Scale-14, n=1; PTSD Symptom Scale-10, n=1

^c^*Inconfort des Patients en REAnimation* (ICU patient discomfort scale), n=2; Short Form-12, n=1

^d^Extended Glasgow Outcome Scale, n=1
